# Is There Still a Role for Succinylcholine in Contemporary Clinical Practice?

**DOI:** 10.31480/2330-4871/100

**Published:** 2019-09-12

**Authors:** Christian Bohringer, Hana Moua, Hong Liu

**Affiliations:** Department of Anesthesiology and Pain Medicine, University of California Davis Health, Sacramento, California, USA

**Keywords:** Succinylcholine, Depolarizing muscle relaxant, Rapid sequence induction

## Abstract

Succinylcholine is a depolarizing muscle relaxant that has been used for rapid sequence induction and for procedures requiring only a brief duration of muscle relaxation since the late 1950s. The drug, however, has serious side effects and a significant number of contraindications. With the recent introduction of sugammadex in the United States, a drug that can rapidly reverse even large amounts of rocuronium, succinylcholine should no longer be used for endotracheal intubation and its use should be limited to treating acute laryngospasm during episodes of airway obstruction. Given the numerous risks with this drug, and the excellent ablation of airway reflexes with dexmedetomidine, propofol, lidocaine and the larger amounts of rocuronium that can now be administered even for an anesthesia of short duration. The use of succinylcholine for endotracheal intubation should disappear from clinical practice.

## Introduction

The depolarizing muscle relaxant drug succinylcholine was introduced in the United Sates in 1952 and has been widely used in clinical medicine since the late 1950s [1,2]. It was initially widely accepted as an intubation drug due to its rapid onset and its short duration of action. A significant advantage of this drug over other muscle relaxants was the short duration of action in most patients as long as the patient did not have a pseudocholinesterase deficiency. Since the introduction of the rapid acting reversal agent sugammadex that is specific for rocuronium this advantage is no longer present [3].

## Disadvantages of Succinylcholine

In 1993 the Food and Drug Administration (FDA) issued a “Black Box Warning” in response to a series of intractable cardiac arrests that occurred in children with previously undiagnosed muscular dystrophy [4]. This warning significantly reduced its use in children and adolescents. Duchenne’s muscular dystrophy was the condition most commonly involved. This is an x-linked disease which explains why pre-pubertal boys were at an especially increased risk of sudden hyperkalemic cardiac arrest following the administration of succinylcholine. In 1993 the package insert was revised to the following: “Except when used for emergency tracheal intubation or in instances where immediate securing of the airway is necessary, succinylcholine is contraindicated in children and adolescent patients…”. There were objections to this package insert by some anesthesiologists and in late 1993 it was revised to “In infants and children, especially in boys under eight years of age, the rare possibility of inducing life-threatening hyperkalemia in undiagnosed myopathies by the use of succinylcholine must be balanced against the risk of alternative means of securing the airway” [5,6]. These hyperkalemic cardiac arrests are not very common due to the relatively low incidence of undiagnosed Duchenne’s and other muscular dystrophies. They are, however, especially traumatic because they occur in apparently healthy children whose myopathy was previously undiagnosed and they frequently end in death of the child or a child with significant brain damage. If a hyperkalemic arrest occurs, calcium as well as insulin and glucose need to be administered [7]. Only about half the patients will survive this type of hyperkalemic cardiac arrest with appropriate treatment and the risk of hyperkalemia should, therefore, not be taken lightly.

### Cardiac arrests

**Figure 1:**
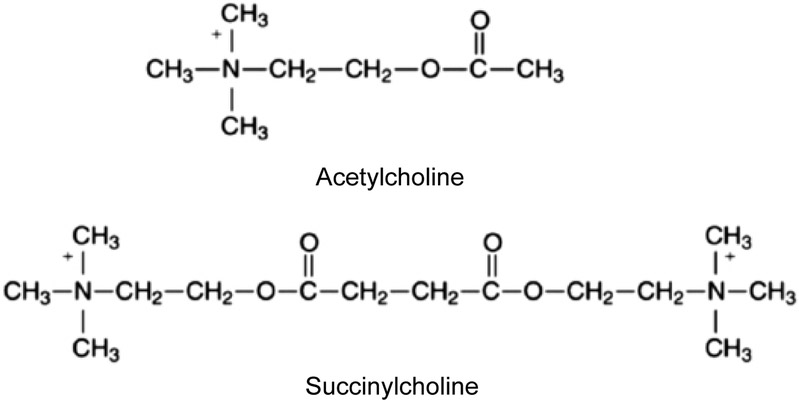
Parasympathomimetic action because it is essentially two molecules of acetylcholine combined with each other.

following succinylcholine occur in both children and adults. There are many cases of hyperkalemic cardiac arrest in patients with stroke, spinal cord injury, burns and immobilization. These conditions are associated with an extra-junctional proliferation of acetylcholine receptors in skeletal muscle which causes an exaggerated release of potassium from skeletal muscle cells following the administration of succinylcholine. It takes some time for this extra-junctional proliferation to occur but hyperkalemia has been observed as early as four days after the injury [8]. Given the high prevalence rate of contraindication to succinylcholine its use in the intensive care unit has been discouraged [9]. In the emergency department, its use is also problematic because there is often only a limited amount of information available about a patient’s health status when an unconscious patient needs to be urgently intubated. Rocuronium is also increasingly replacing succinylcholine in the prehospital environment [10] Succinylcholine is also associated with the risk of severe bradycardia and asystolic cardiac arrest. This is due to its parasympathomimetic action because it is essentially two molecules of acetylcholine combined with each other (Figure 1). This is more likely to occur with large and repeat doses, and succinylcholine infusions. When large doses or an infusion are administered the patient should be pre-treated with a prophylactic dose of atropine prior to administering the succinylcholine. Infusions of succinylcholine in particular are an outdated anesthesia technique that should no longer be employed because of the risk of bradycardia and prolonged duration of neuromuscular block.

### Prolonged neuromuscular block:

Recovery from neuromuscular blockade is unpredictable after succinylcholine. The prevalence of pseudocholinesterase deficiency is much higher than that of undiagnosed myopathies. Significantly prolonged apnea following succinylcholine occurs in around one in three thousand administrations [11]. The cause is usually a congenital deficiency of the pseudocholinesterase enzyme but severe liver disease and malnutrition may also lead to prolonged paralysis [12,13]. Patients who experience a prolonged block after succinylcholine will also have a prolonged block after mivacurium because this drug is also eliminated by the same plasma enzyme [14]. Butyryl-cholinesterase deficiency is a condition characterized by autosomal recessive inheritance and novel mutations in the butyryl-cholinesterase gene continue to be identified [15].

Unlike the cardiac arrests that occur with myopathies and immobilization, the prolonged neuromuscular block in patients with pseudocholinesterase deficiency is not fatal as long as the persistent weakness is recognized and appropriately treated prior to extubation. Clinical signs of persistent neuromuscular block are: a lag of the eye lids when trying to open the eyes and lack of antigravity power with the classic “fish out of water” appearance need to be recognized before extubation. If the patient is extubated before the succinylcholine has worn off this will lead to hypoxia that is followed by an emergency re-intubation. The pupils are usually dilated and the patient is tachycardic and hypertensive because being awake with persistent neuromuscular weakness leads to a potent activation of the sympathetic nervous system. It is important to check the pupil diameter because persistent neuromuscular block is often misdiagnosed as delayed emergence from general anesthesia drugs. If excessive amounts of general anesthesia drugs are postulated to be the cause for a patient’s non-responsiveness after an anesthesia, then the pupils should be constricted. A nerve stimulator can be used to distinguish a depolarizing block without fade on train of four (TOF) and without post-tetanic facilitation due to succinylcholine from a non-depolarizing block with fade on TOF and post-tetanic facilitation due to residual rocuronium, vecuronium or cis-atracurium. It is, however, very important in this circumstance to administer some propofol to the patient prior to the use of the nerve stimulator to prevent recall of the tetanic stimulus. It is already stressful enough for a patient to be awake and feeling weak. An awake patient with persistent neuromuscular block should never be subjected to the painful stimuli of a nerve stimulator without proper sedation [16]. The patient should be reassured that the weakness that he/she feels is a side effect of a medication and will get better. Many patients with a persistent neuromuscular block after anesthesia think that they had a stroke during their surgery and an explanation and reassurance to them while they are still intubated is of utmost importance. If there is fade and post-tetanic facilitation the residual neuromuscular block may be due to rocuronium, then, more sugammadex should be administered to reverse it. A non-depolarizing block may also be due to a phase two block in patients that have a butyryl-cholinesterase deficiency or have been administered a large dose of succinylcholine especially by infusion [17,18]. If fade on TOF and post-tetanic facilitation are absent or a nondepolarizing block with rocuronium does not reverse with sugammadex, the persistent neuromuscular block is due to succinylcholine and the patient most likely has a pseudocholinesterase deficiency [19]. In this case, it may take several hours for the neuromuscular block to wear off. A plasma pseudocholinesterase level should be sent to confirm the diagnosis. The dibucaine number helps to distinguish genetic causes of butyryl-cholinesterase deficiency. Homozygotes will have a dibucaine number < 20, heterozygotes 40-70 and patients with normal enzyme levels > 70. Recombinant pseudocholinesterase can reverse succinylcholine induced apnea [20]. This recombinant enzyme is generally not available in hospitals for use in humans. Two units of fresh frozen plasma (FFP) can be administered to reverse the block because this amount of FFP contains sufficient enzyme to reverse the block [21] FFP is usually not given because of the risk of infection and fluid overload. This usually leads to the patient having to be ventilated for several hours in an intensive care unit. The use of succinylcholine should be especially avoided in free-standing surgical centers where intensive care admission would require transfer of the patient to another hospital. If pseudocholinesterase deficiency is confirmed the patient should be advised to warn future anesthesiologists of this and succinylcholine and mivacurium should be avoided in the future. It is also useful to test the relatives for pseudocholinesterase deficiency. While residual neuromuscular blockade from excess rocuronium can now be easily corrected with further administration of sugammadex there is, unfortunately, at the moment no readily available antidote to succinylcholine that is devoid of side effects.

### Intracranial pressure:

Raised intracranial pressure in response to succinylcholine can be mitigated by a defasciculating dose of non-depolarizing NMBA and by normalizing the partial pressure of carbon dioxide in the arterial blood following intubation. A recent re-publication of a landmark article noted however a 100% increase in intracranial pressure in cats following succinylcholine and concluded that succinylcholine may be contra-indicated in patients with raised intracranial pressure [22]. Raised intraocular pressure cannot be reliably abolished by de-fasciculation and succinylcholine is relatively contraindicated in patients with open eye injury. If succinylcholine is to be used in patients with open eye injury pre-treatment with a defasciculating dose of non-depolarizing neuromuscular blocking drug should be used [23,24].

### Masseter spasm

after succinylcholine makes endotracheal intubation difficult or impossible and may herald the development of a malignant hyperthermia reaction. If masseter spasm occurs the patient needs to be carefully monitored for increased carbon dioxide production and the development of hyperthermia [25,26]. When masseter spasm is severe it presents a difficult airway management problem and there is a case report of succinylcholine induced masseter spasm during attempted rapid sequence intubation requiring a surgical airway [27].

### Succinylcholine and malignant hyperthermia:

Unlike the non-depolarizing NMDA, succinylcholine is a potent trigger for malignant hyperthermia and it is absolutely contraindicated in patients at risk of MH (Table 1). It has been estimated that the use of succinylcholine increases the risk of developing a malignant hyperthermia reaction under volatile anesthesia by a factor of twenty [28].

### Anaphylaxis and allergic reactions:

Muscle relaxants are the most common cause of intra-operative anaphylaxis. The incidence of anaphylaxis with succinylcholine is only a little bit higher than with rocuronium [29,30]. Unlike with rocuronium, there is no binding agent available for succinylcholine to sequester it out of circulation. There have been several case reports in the literature which report a significant improvement in the severity of the anaphylactic reaction to rocuronium following the administration of sugammadex [31-34]. Some laboratory studies do not support the hypothesis that rocuronium induced anaphylaxis can be ameliorated by sugammadex [35]. A case control study also found an inconsistent response to sugammadex on rocuronium induced anaphylaxis [36]. This may have been due to an inadequate amount of sugammadex used. When seeking to ameliorate an anaphylactic response to sugammadex a dose of at least 16 mg/kg of sugammadex is recommended to ensure that all the rocuronium can be encapsulated by the cyclodextrin [34].

### Inadequate relaxation:

There are some instances where there seems to be an inadequate relaxation response to succinylcholine. This may occur in a patient with undiagnosed myotonic dystrophy which is an autosomal dominant inherited condition with a prevalence of about 1 in 8,000 people. In patients with myotonic dystrophy succinylcholine causes a paradoxical contractile response. The clinical signs of myotonia congenita are frontal baldness, hypertrophy of distal muscle groups in the limbs and delayed relaxation of the grip during a hand shake. The myotonia frequently has not been clinically diagnosed before the operation because these signs are often very subtle. The anesthesia care provider may therefore unexpectantly encounter a patient in trismus and myotonia following succinylcholine [37]. The myotonic contractile response to succinylcholine can then be abolished by administering a non-depolarizing NMBA [38]. Lack of a relaxant response to succinylcholine can also occur when to the drug has lost its potency from having been stored out of the refrigerator for too long. Succinylcholine should be stored in the refrigerator at 2-8 °C to prevent loss of potency as recommended by the manufacturer. It will lose 2% of its potency per month at room temperature and 8% per month when stored at 37 °C [39].

Sometimes there are multiple adverse reactions with succinylcholine in the same patient. A case of anaphylaxis to succinylcholine during caesarian section together with prolonged paralysis from pseudocholinesterase deficiency in the same patient has been reported in the literature [40]. Myalgias are another common problem and they cannot reliably be abolished by a defasciculating dose of NMBA [41]. The cost of all the problems associated with succinylcholine (Table 1) is a lot higher than its acquisition cost. In a pharmaco-economic study the true cost to society of succinylcholine has been estimated to be around twenty times the acquisition cost when instances of death or severe brain injury from the use of this drug are taken into account [42].

## Intubating Conditions with Rocuronium during Rapid Sequence Induction

Succinylcholine is no longer necessary for rapid sequence induction because the rapid onset of neuromuscular block with a dose of 1.2 mg of rocuronium/kg results in excellent intubating conditions [43]. A recent meta-analysis found no difference in intubation conditions when comparing succinylcholine to rocuronium [44]. In the emergency department succinylcholine and rocuronium were equivalent with regard to first attempt intubation success [45]. A national emergency airway registry study comparing intubation success with succinylcholine versus rocuronium found no association between paralytic choice and first pass rapid sequence intubation success or peri-intubation adverse events [46]. An alternative to using a large dose of 1.2 mg/kg rocuronium is the use of a priming dose that results in satisfactory intubating conditions for rapid sequence induction with only 0.6 mg/kg [47]. Intravenous induction agents like propofol, dexmedetomidine and lidocaine also ablate the gag reflex very well [48-51]. This allows for good intubating conditions even before the rocuronium has reached its maximal effect. The absence of respiratory depressant effects of dexmedetomidine and lidocaine also make these drugs ideal agents for co-induction during a rapid sequence induction with propofol because they do not impede pre-oxygenation like all the opioid drugs with their profound respiratory depressant effects. Propofol ablates the gag reflex much better than thiopental and this explains why studies comparing succinylcholine with rocuronium found better intubating conditions with succinylcholine when thiopental was the induction agent that was used [52]. In studies employing propofol as the induction agent intubating conditions with rocuronium were equivalent to succinylcholine. Propofol should therefore be preferred over thiopental for rapid sequence induction with rocuronium to provide for better intubating conditions (Table 2). If endotracheal intubation cannot be achieved and it is decided to wake the patient up again rocuronium is also a safer agent because reversal of profound high-dose rocuronium-induced neuromuscular block (1.2 mg/kg) with 16 mg/kg sugammadex is significantly faster than spontaneous recovery from 1 mg/kg succinylcholine [53,54].

## Disadvantages of Using Rocuronium and Sugammadex in Rapid Sequence Induction

To present a balanced view on this topic the disadvantages of the rocuronium and sugammadex combination need to be discussed. Rocuronium has a higher rate of anaphylaxis (1:2,500) than vecuronium or cis-atracurium (1:22,000) but not higher than succinylcholine (1:2,000) [29]. The severity of the anaphylactic reactions is the same with the different relaxants [55]. When replacing succinylcholine with rocuronium the anaphylaxis risk of the sugammadex needs to be added to the anaphylaxis risk of the muscle relaxant. In many cases when rapid sequence induction is employed however non-depolarizing neuromuscular blockade is established when the succinylcholine has worn off to improve operating conditions for the surgeons. In this common scenario the patient is then exposed to the anaphylaxis risk of two muscle relaxants as well as the reversal agent. The anaphylaxis incidence with sugammadex has been estimated to be about 1: 35,000 cases [30]. The incidence has also been reported to be higher with larger doses of the drug [56]. Anaphylaxis to sugammadex can occur in healthy patients that had no prior exposure to it [57]. The complete elimination of the rocuronium-sugammadex complex remains poorly understood in renal impairment and at present its use in patients with severe renal failure is therefore not recommended. Information on its use in pediatric patients less than two-years-old is also still limited. Rocuronium is associated with a 2% incidence of hypotension or hypertension and in some patients this may be significant.

## Current Role of Succinylcholine

The current role of succinylcholine should be as a stand-by drug to treat laryngospasm. Small doses of around 10 mg that are small enough to break the laryngospasm but not large enough to cause laryngeal or diaphragmatic weakness are usually administered. This will treat the laryngospasm without requiring the patient to be ventilated or intubated. With the help of sugammadex however an excessive dose of rocuronium can now be rapidly reversed and it is conceivable that in the future rocuronium will take over the role of the stand-by drug to treat laryngospasm as long as the patient has an intravenous catheter *in situ*. In a study of patients with burn injury a dose of 1.5 mg/kg of rocuronium produced an initial onset of paralysis as early as 30 seconds which the authors felt was a reasonable onset time for the relief of laryngospasm [58]. This may be fortuitous because succinylcholine loses a significant amount of its activity when it is stored outside a refrigerator and it may not work if it has been stored at room temperature for too long. This is likely to occur with any medication that needs to be always readily available in the operating room but is almost never used. During inhalational induction, when there is no intravenous catheter *in situ* it is still prudent to treat laryngospasm with intramuscular or sublingual succinylcholine because of its faster onset when given intramuscularly.

## Summary

Succinylcholine has a lot of side effects and is associated with a lot of adverse events. Rocuronium has fewer side effects and with the availability of sugammadex reversal of neuromuscular block can be achieved more rapidly and reliably than spontaneous recovery after succinylcholine. Succinylcholine should no longer be used for rapid sequence induction or for cases that are thought to be of short duration when rocuronium is readily available, there is no concern for allergic reaction to rocuronium and no contraindication to non-depolarizing neuromuscular blockade exists. Use of succinylcholine should be limited to treat acute laryngospasm and it should no longer be used as an intubation drug when the above criteria are met.

## Figures and Tables

**Table 1:** Disadvantages of Succinylcholine.

May induce hyperkalemic cardiac arrest in children with unrecognized myopathy and patients with stroke, spinal cord damage, burns, immobilization or known myopathy
Risk of severe bradycardia or asystole when with large, repeat doses or infusions
Prolonged duration of action in patients with pseudo-cholinesterase deficiency
Myalgias
Raised intracranial pressure
Raised intraocular pressure
Masseter spasm
Risk of malignant hyperthermia
Lack of efficacy when succinylcholine has been out of the refrigerator for too long
No reversal agent available clinically except for fresh frozen plasma

**Table 2:** Advantages of rocuronium over succinylcholine.

Faster reversal of neuromuscular blockade with sugammadex than spontaneous recovery after succinylcholine
No myalgias
No masseter spasm or malignant hyperthermia
No raised intracranial and intra-ocular pressure
